# Investigation of novel passive methods of generation of swirl flow in supersonic separators by the computational fluid dynamics modeling

**DOI:** 10.1038/s41598-022-18691-x

**Published:** 2022-08-24

**Authors:** Sina Nabati Shoghl, Abbas Naderifar, Fatola Farhadi, Gholamreza Pazuki

**Affiliations:** 1 Department of Chemical Engineering, Amirkabir University of Technology (Tehran Polytechnic), Tehran, Iran; 2 Department of Chemical and Petroleum Engineering, Sharif University of Technology, Azadi Ave., Tehran, Iran

**Keywords:** Chemical engineering, Mechanical engineering

## Abstract

In this paper, three passive methods for the generation of swirl flow in the supersonic separator (3S) were investigated, and their structures were optimized by computational fluid dynamics (CFD) modeling. The influence of the structural and operational parameters on the dew point depression, phase envelope diagram, rate of natural gas liquid (NGL) recovery, and separation efficiency have also been evaluated. The collection efficiency was significantly improved for the nozzle equipped with the passive swirler compared with the simple nozzle. The selection of passive swirler type played a crucial role in the natural gas liquefaction and separation. The side injected swirler, and serpentine swirler showed the most significant improvement in separation efficiency than the U-turn swirler. For the side injected swirler at the optimum injection angle, the maximum collection efficiency was about 89% at the pressure loss ratio (PLR) of 0.2. Besides, the simulation results demonstrated that for the serpentine 3S, with the increase in serpentine twist number, the highest improvement on the collection efficiency of the investigated nozzle was obtained. In addition, it was observed that, when the convergent section profile was designed according to the Witoszynski line-type, a larger refrigeration zone was obtained than other considered profiles.

## Introduction

The extracted natural gas from underground reservoir contains heavy hydrocarbons and water vapor. Correction of water and hydrocarbon (HC) dew points are essential as the presence of liquid phases enhance the risk of pipeline corrosion and hydrate formation during natural gas transportation. Various traditional techniques, including membrane separation, cryogenic process, absorption, and adsorption are employed to correct water and HC dew point^1–4^. These conventional separation methods require large separation equipment, high operating and capital cost and may cause operational problems. In conclusion, a new separation method has to be developed to correct the natural gas dew point. The 3S is a revolutionary method for natural gas dehydration and NGL recovery. The 3S is capable of correction of water and HC dew points with the maximum rate of NGL recovery and minimal cost^3^. The 3S is appropriate for unmanned operations, especially for remote areas and offshore plants^5^. The separation process inside the 3S takes about two milliseconds^6^. Consequently, this novel technology does not require chemical inhibitors to prevent hydrate formation due to the very low residence time of the separation process.

In recent years, a significant part of the studies have been done to optimize the thermal efficiency of various processes^3,7–9^. Process simulations and numerical studies are generally used for these optimizations^4,10–12^. The thermal efficiency can be improved in two ways: 1—modification of the mechanical structure of the investigated equipment^7^ and 2—improvement of the thermo-physical properties of the base fluid^13–15^. For example, Ulah et al.^14^ employed the hybrid nano-liquid including a combination of AA7072 and AA7075 nanoparticles and water (water-aluminum alloy nanoparticles) to improve the heat transfer of investigated fluid. They observed great influence for hybrid nano-materials throughout their study compared to the regular nanomaterials. In another work, Ulah et al.^16^ used two types of nanofluids including multi-walled and single-walled carbon nanotubes as nanoparticles and ethylene glycol as a base fluid. They observed that the Nusselt number is higher for single-walled carbon nanotubes compared to the multi-walled carbon nanotubes. They attributed this behavior to the higher thermal conductivity of multi-walled carbon nanotubes compared to the single-walled carbon nanotubes.

Three main refrigeration processes include Joule–Thomson (JT) process, turbo-expander process and 3S process^3^. The JT process is the most common liquefaction process in the natural gas industry, however, based on the results the separation efficiency of 3S is higher than JT process^3,7^. Alfyorov et al.^17^ reported this converging–diverging nozzle separates the natural gas species with up to about 20% saving in compressor energy consumption compared to the same operational condition conducted by the JT valve and Turbo-expander. The 3S has high availability due to the lack of moving parts. The first group working on this technology was an engineering team from the Netherlands that is called the Twister BV^18^. At the same time, a Russian engineering team also studied the industrial application of this equipment and called it 3S technology^17^. Previously three different methods were proposed for generation of swirl flow inside the nozzle. One method is implementing the U type supersonic separator^19,20^, the second is using the delta wing which is mounted after the nozzle outlet^21^ and the other is utilizing the static vane for generation of swirl flow^7^.

In the last few years, the use of mathematical modeling^22–24^ and CFD modeling^3,25^ in explaining the behavior of various physical phenomena has increased significantly. CFD modeling was the primary method in the evaluation of the flow behavior through the 3Ss. For example, Jassim et al.^26,27^ employed the CFD modeling to investigate the flow properties of high-pressure natural gas in the 3S. They investigated the influence of vorticity, real gas properties, and nozzle structure on the performance of the 3S. They observed that the location of the shockwave significantly changed by assuming a real gas model instead of the perfect gas model. Haghighi et al.^20^ suggested a novel U-turn 3S for the generation of centrifugal force and simulated the proposed structure with the CFD modeling. They reported that the CFD modeling is a valuable and reliable tool for analyzing 3S’s behavior. Yang and Wen^28^ used the CFD modeling to investigate the particle motion behavior inside the 3S with the strong swirl. They observed that when the size of the liquid droplets became larger than 1.5 µm, the separation efficiency over 80% could be obtained. Escue^29^ employed two turbulence models, including the Reynolds stress model (RSM) and the RNG κ-ε model, to simulate numerically swirl flow inside a straight pipe. The results illustrated that at high swirl flow, the RSM model more accurately predicted the experimental velocity profile, while at low swirl flow, the RNG κ-ε model better predicted the experimental values. Liu et al.^30^ employed the Discrete Particle Method (DPM) to predict the droplet behavior inside a converging–diverging nozzle where the droplet diameter varies in the range of 10–50 µm. In several papers, the influence of operational parameters on the separation efficiency was also predicted by the CFD modeling. For instance, Bian et al.^31^ simulated the supersonic separation with CFD modeling to analyze the condensation of the natural gas using this equipment. They investigated the influence of component composition, outlet pressure, feed temperature, and pressure on the condensation process. Vaziri and Shahsavand^32^ optimized the natural gas velocity components using COMSOL software to maximize both centrifugal acceleration and swirl strength. Finally, the influence of inlet pressure and temperature on the centrifugal acceleration and swirl number were also studied.

In the last years, several experimental and numerical studies had been conducted to investigate the influence of structural parameters on the separation behavior of the 3S. For instance, Wen et al.^33^ studied numerically the flow field and particle separation characteristics by the DPM in the 3S. They observed that the geometry of the nozzle could improve the separation performance. The structure of the 3S was optimized by Bian et al.^34^. They postponed the generation of shockwave by decreasing the opening angle of the expanding section. This modification enhanced the domain of the low-temperature region and improved the separation efficiency and the refrigeration performance of the 3S. Wen et al.^35^ investigated numerically the natural gas turbulence flow in three newly designed diffusers. They reported that the conical diffuser with high-pressure recovery was a suitable case for the converging–diverging nozzle. The Laval nozzle played a crucial role in the liquefaction of condensable components in the supersonic separation method. Zhao et al.^36^ numerically studied the influence of the line-type of the convergent section on the condensation flows process of superheated steam in a 3S. They observed that a slow shrink of the convergent profile postponed the formation of liquid droplets. Hou et al.^37^ designed the convergent part of 3S using four line-types including Bi-cubic curve, Quintic polynomial curve, Witoszynski curve, and translation of Witoszynski curve. They reported that the number of droplets had a tendency to enhance for the convergent section designed by the translation of Witoszynski curve. The influence of operational parameters such as inlet temperature, inlet pressure, back pressure, and mass flow rate has also been studied in several works^38,39^. For example, Liu et al.^40^ investigated a natural gas dehydration unit and observed that for an indoor 3S rig, the fluid flow rate, shockwave position, and PLR had a significant influence on the unit dehydration behavior. The influence of several parameters including inlet temperature, inlet pressure, inlet velocity, outlet velocity, and pressure recovery, on the size and structure of the 3S was also evaluated by Vaziri and Shahsavand^41^ using a trained neural network. Niknam et al.^42^ developed a neural network model for the characterization of flow behavior inside the 3S. They reported that the sensitivity of shock wave position to pressure boundary condition was higher than temperature boundary condition.

The 3S can be used in the oil and gas industry to correct HC and water dew points, improve NGL recovery and dehydrate natural gas. This paper focused on employing the 3S to increase the NGL recovery of an existing JT process. The objective of this study is optimization of the 3S configuration and swirler to improve the separation performance with suitable pressure recovery. To achieve this, an alternative passive method for generation of swirl flow inside the 3S was presented. The cooling performance and collection efficiency were employed to characterize the separation behavior inside the 3Ss. Several new types of swirler with a novel geometry, including side injected, serpentine and U-turn swirler, had been considered, then, the optimal structure and operational condition required to generate swirl flow inside the 3S were found by the CFD modeling. These 3Ss operated under passive mode. To the best of the author’s knowledge, the side injected and serpentine swirler for the generation of swirl flow inside the 3S was not reported so far.

## Mathematical modeling

**Figure 1:**
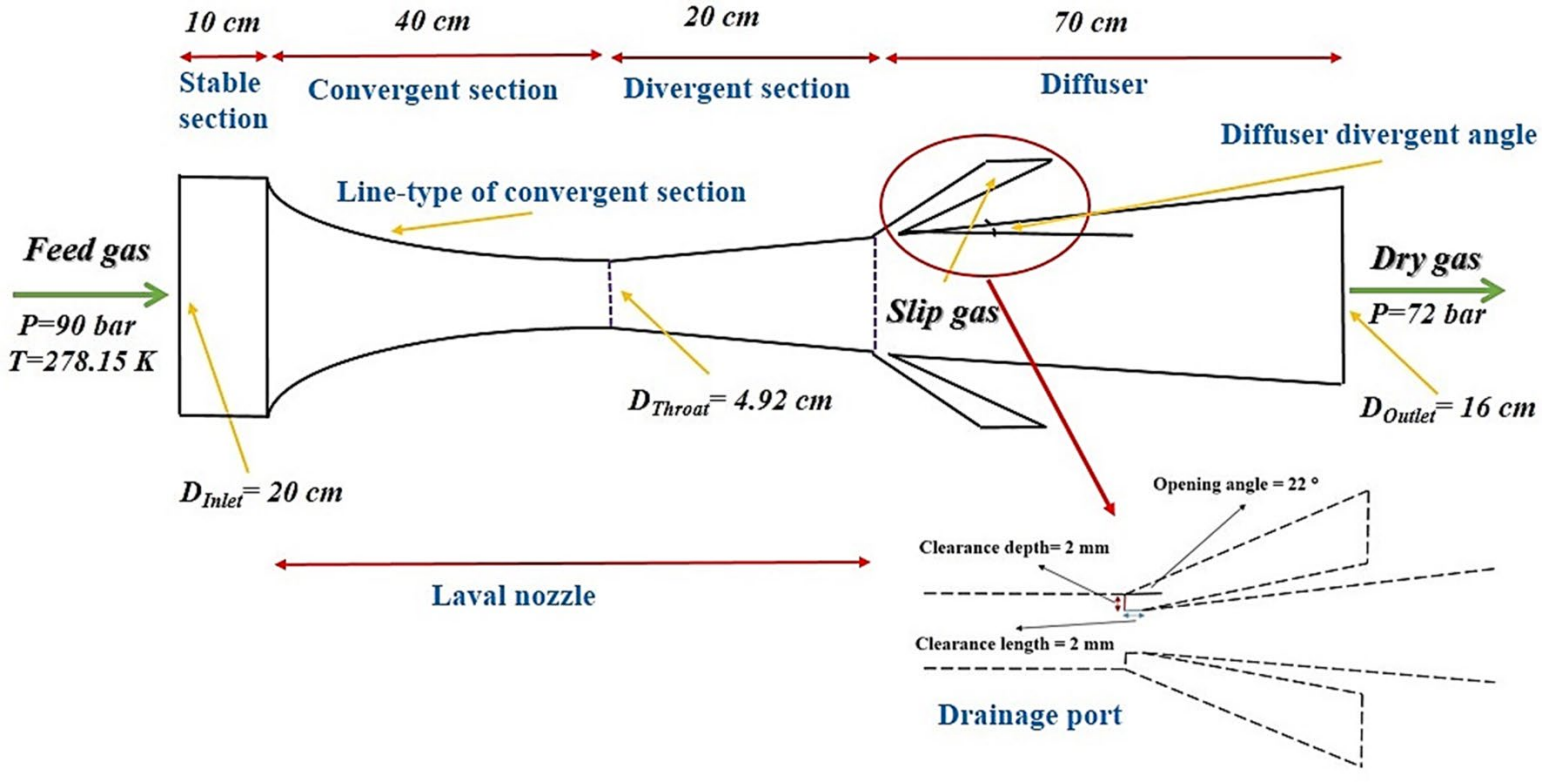
Schematic of the studied 3S.

In this study, a comparative investigation was conducted to analyze the influence of using passive methods for the generation of swirl flow on the nozzle performance. In the last years, two methods, including the process simulation software and the CFD modeling, were used for predicting the separation efficiency of the 3S^3^. In this study, firstly, the CFD modeling was used to select the suitable structure of the passive swirler. Then, the Aspen Plus simulation software was employed to determine the phase envelope diagram, rate of NGL recovery, and the dew point depression for the optimized structure. A 3S is composed of different sections, including swirler, Laval nozzle, drainage port, and diffuser (Fig. 1). The Laval nozzle is the main part of a converging–diverging nozzle which includes a convergent part, a throat section, and a divergent section. In the Laval nozzle, the natural gas is expanded, its velocity enhanced and the gas cooled, and the liquid droplets are initiated and formed. After that, the centrifugal flow generated by a swirler directed the liquid droplets toward the separator wall. The liquid phase will be separated at the drainage port, and dry gas will be directed toward the gas outlet. The opening angle of 22°^43^ was selected for the drainage port. The role of the diffuser is to recover the pressure. The detailed dimensions of the 3S are presented in Fig. 1. In the entrance of the Laval nozzle, a stable section was installed to make the feed gas flow uniform and less turbulent. The suitable length of the stable section was about 0.5 *D*_*in*_^44^.

The natural gas velocity is increased through the convergent section from the inlet to the throat, but the highest velocity was obtained at the throat. If the gas velocity was sonic at the throat, it is enhanced to supersonic at the divergent section of the Laval nozzle. However, if the gas velocity was subsonic there, it will be subsonic through the total length of the nozzle. After the throat, depending on the structure of the divergent section, a sudden variation in temperature, pressure, and velocity will be observed. The Mach number is a dimensionless parameter, which is defined as follow:

1 $$Ma = \frac{u}{C}$$

where *u* is the gas velocity inside the nozzle and C is the sound speed which is calculated by the following equation^45^:

2 $$C = \sqrt {\left( {\frac{\partial P}{{\partial \rho }}} \right)_{S} } = \sqrt { - V\left( {\frac{\partial P}{{\partial V}}} \right)_{S} }$$

where *V* is the specific volume of the natural gas. The natural gas flow behavior in the 3S was described by the mass, momentum, and energy equations, respectively. These equations are represented by Eqs. (3)–(6).

The mass conservation equation is:

3 $$\frac{\partial \rho }{{\partial t}} + \frac{{\partial \left( {\rho u} \right)}}{\partial x} = 0$$

The momentum conservation equation is:

4 $$\frac{\partial }{\partial t}\left( {\rho u} \right) + \nabla \left( {\rho uu} \right) = - \nabla P + \nabla \left( r \right)$$

where the stress tensor (*r*) is defined by Eq. (5):

5 $$r = \mu \left[ {\left( {\nabla u + \nabla u_{{}}^{T} } \right) - 2/3\nabla uI} \right]$$

The conservation equation of energy is described by Eq. (6):

6 $$\rho C_{p} {{DT} \mathord{\left/ {\vphantom {{DT} {Dt = k\nabla^{2} T}}} \right. \kern-\nulldelimiterspace} {Dt = k\nabla^{2} T}} + Q + Q_{p} + Q_{vd}$$

where T*, t, k, ρ, µ, P, u*, *Q*_*vd,*_,*and Cp,* are temperature, time, thermal conductivity, density, viscosity, pressure, velocity vector, viscous dissipation, and heat capacity at constant pressure, respectively and *Q* is the heat source. Additional terms, including pressure work (*Q*_*p*_) (Eq. (7)) and viscous dissipation (*ϕ*) (Eq. (8)), were imported into Eq. (6) to calculate the temperature drop through the 3S^46^:

7 $$Q_{P} = \alpha_{P} Tu \cdot \nabla P_{A}$$

8 $$\phi = 2\mu \left[ {\left( {\frac{\partial u}{{\partial x}}} \right)^{2} + \left( {\frac{\partial v}{{\partial y}}} \right)^{2} + \left( {\frac{\partial w}{{\partial z}}} \right)^{2} + \frac{1}{2}\left( {\frac{\partial v}{{\partial x}} + \frac{\partial u}{{\partial y}}} \right)^{2} + \frac{1}{2}\left( {\frac{\partial w}{{\partial y}} + \frac{\partial v}{{\partial z}}} \right)^{2} + \frac{1}{2}\left( {\frac{\partial u}{{\partial z}} + \frac{\partial w}{{\partial x}}} \right)^{2} } \right]$$

where:

9 $$\alpha_{P} = - \frac{1}{\rho }\left( {\frac{\partial \rho }{{\partial T}}} \right)_{P}$$

The natural gas analysis from an NGL recovery plant was selected for this study. The considered natural gas composition (mole %) (From Gas Chromatography analysis) is presented in Table 1.

**Table 1:** The natural gas composition.

Component (mole %)	CH_4_	C_2_H_6_	C_3_H_8_	i-C_4_	n-C_4_	i-C_5_	n-C_5_	C_6_H_14_	C_7_+	CO_2_	N_2_	H_2_O
Composition	89.4	3.7	1.55	0.31	0.42	0.1	0.07	0.04	0.01	1.1	3.3	0.03

### Type of swirl generator

One of the main parts of the 3S is the swirler. In this study, several new geometries were considered to investigate the effect of modifying the structure of the swirler on the swirl strength and separation efficiency. Most of the previously published works have employed an active swirler for the centrifugal force generation^47,48^. For the conventional active swirler, to generate the swirl flow, a set of static vanes were mounted on a conical internal body^47,48^. In order to improve the cooling performance and consequently increase the production rate of gas condensate, new configurations should be proposed for the swirler. Therefore, the effect of using three passive swirler on the 3S performance was further evaluated. Generally, there are two locations for the installation of a swirler: (a) at the nozzle entrance and (b) after the Laval nozzle. Table 2 presents the considered passive swirler in this study.

**Table 2:**
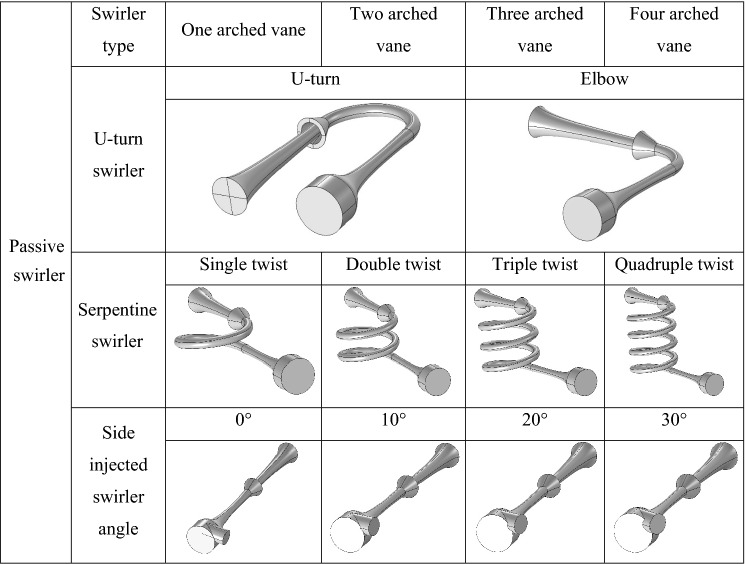
Structure of various investigated passive swirler.

#### Practical application of 3S equipped with passive swirler

Compared to the traditional separation process, the 3S can provide higher separation performance, lower size, compact design, higher availability and no chemical consumption^3,7,41^. In addition, the 3S can be employed for sweetening of sour natural gas and correction of water and HC dew point^3,7,49^. In this study, the passive method was employed to generate the swirl flow which means any external equipment was not used to generate the centrifugal force. In another words, the passive method utilizes no additional device but the feed gas energy was used for generation of swirl flow. The passive swirler not only reduces the energy loss, but also generates strength swirl flow for separation of liquid droplets. Therefore, more energy is available to lower the minimum temperature. The required temperature to liquefy the light hydrocarbons cannot be obtained by the JT processes, whereas, the optimized 3S can provide high ethane and propane recovery due to the very low temperature achieved inside the Laval nozzle^3,7^. Therefore, the 3S designed based on the passive swirler not only can be used instead of the JT process for NGL recovery (described in “Considered NGL recovery unit” section), but it can also be used for sweetening^49^ and dehydration of natural gas^50^.

### Particle tracing model

In this study, the gas phase and the liquid droplets were considered as the continuous phase and dispersed phase, respectively. The density of water droplets and condensate droplets were considered about 994.4 kg/m^3^ and 584.6 kg/ m^3^, respectively. A spherical shape with uniform distribution was assumed for the liquid droplets. The typical droplet size in the 3S was about 0.1–2 µm^51^. In addition, the particle tracing model was used to track the motion of the liquid droplets inside the 3S and evaluate the influence of liquid droplet size on the separation efficiency. In a 3S, the liquid droplet motion was tracked by force balance on a droplet. Another vital force inserted on a droplet in the gas flow is the drag force. The Schiller-Neuman^52^ equation was employed to consider the inserted drag force on the droplets. This force was defined by Eq. (10):

10 $$F_{D} = \frac{{3\mu C_{D} {\text{Re}}_{r} }}{{4\rho_{p} d_{p}^{2} }}mp\left( {w^{\prime} - u} \right)$$

where $w^{\prime}$ and *C*_*D*_ (drag coefficient) were determined by Eqs. (11) and (12), respectively^52^:

11 $$w^{\prime} = u + \Delta u \quad \Delta u = \xi \sqrt{\frac{2k}{3}}$$

12 $$C_{D} = \frac{24}{{{\text{Re}}_{r} }}\left( {1 + 0.15\left( {\frac{{\rho \left| {u - v} \right|d_{p} }}{\mu }} \right)^{0.687} } \right)$$

where *ξ* was the un-correlated Gaussian number vector. The mutual interaction between droplets was neglected in this study. Furthermore, for the considered droplets, due to the larger induced centrifugal than gravity force, the effect of the latter could be neglected. The collection efficiency was one of the most crucial parameters in evaluating a convergent-divergent nozzle’s performance. In this work, the collection efficiency was calculated based on the number of the separated droplets in the 3S. The collection efficiency is defined by Eq. (13):

13 $$N = \frac{{n_{Inlet} - n_{Outlet} }}{{n_{Inlet} }} \times 100\%$$

where *n*_*Inlet*_ is the number of injected droplets and *n*_*Outlet*_ is the number of droplets carried by the gas phase.

### Boundary condition and pressure loss ratio

The pressure boundary condition was specified for the inlet and outlet of the nozzle. In addition, no-slip and adiabatic boundary conditions were considered for the separator walls. The inlet temperature, inlet pressure, and outlet pressure were equal to 278.15 K, 90 bar, and 72 bar, respectively. Furthermore, the volumetric flow rate of the considered gas plant was about 4 million metric standard cubic meters per day (MMSCMD). The pressure loss ratio (PLR) was defined by Eq. (14):

14 $$\gamma = \frac{{P_{in} - P_{out} }}{{P_{in} }} \times 100\%$$

where *P*_*in*_ and *P*_*out*_ are the pressure of natural gas at the inlet and outlet of the 3S, respectively. In this paper, the PLR of 0.2 was considered for the optimization. The condensation occurs when the natural gas pressure is lower than the cricondenbar and enough time for phase change is available. The temperature drop through the 3S caused the heavy hydrocarbon to liquefy, resulting in natural gas with corrected dew point and different composition. To characterize the separation efficiency, two additional terms, including “dew point depression” and “collection efficiency of each component” were also considered. The dew point depression was expressed by Eq. (15):

15 $$\Delta T_{d} = T_{d}^{in} - T_{d}^{out}$$

where $T_{d}^{in}$ and $T_{d}^{out}$ represented the inlet dew point and outlet dew point of the natural gas. Moreover, the collection efficiency of each component was defined by Eq. (16):

16 $$\eta = \frac{{x_{v}^{in} - x_{v}^{dry} }}{{x_{v}^{in} }} \times 100\%$$

where $x_{v}^{in}$ is the mole fraction of component i at the inlet, and $x_{v}^{dry}$ is the mole fraction component i at the outlet. The dimensionless mass flow rate was also defined by Eq. (17):

17 $$M = \frac{{m_{j} }}{{m_{0} }}$$

where *m*_*0*_ is the mass flow rate through the simple nozzle (3S without any swirler) and *m*_*j*_ is the mass flow rate through the nozzle equipped with swirler.

The cooling performance of each considered structure was calculated by Eq. (18):

18 $$J = \frac{{T_{inlet} - T_{\min } }}{{T_{inlet} - T_{\min - overall} }} \times 100\%$$

After calculating the cooling performance, by multiplying the collection efficiency (*N*) by the cooling performance (*J*), the separation efficiency (*ψ*) was obtained as follows:

19 $$\psi = N \times J$$

where *J, T*_*min-overall*_*, T*_*inlet*_, and *T*_*min*_ are the cooling performance, the minimum temperature inside the optimized nozzle (without swirler), inlet temperature, and the minimum temperature inside the modified nozzle, respectively.

### Selection of the accurate turbulence model for the description of supersonic flow

**Figure 2:**
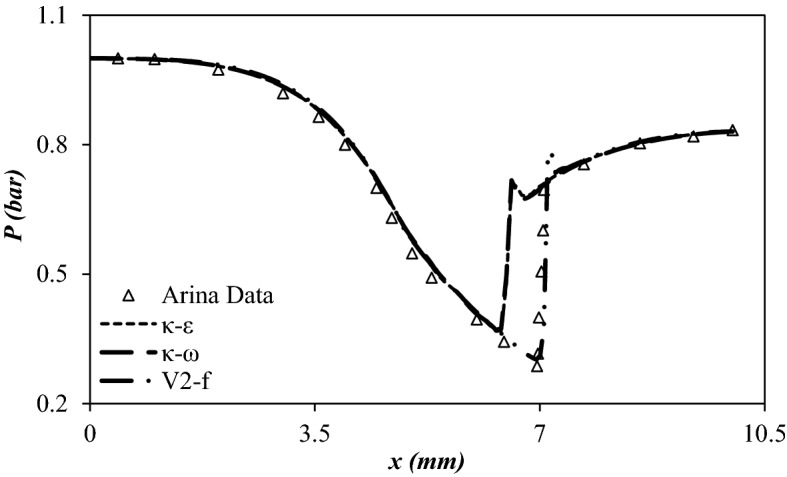
Comparison of predicted profile of various turbulence models with Arina’s data^56^.

Three Reynolds Average Navier–Stokes (RANS) turbulence models were selected to compare their ability for description of fluid flow in the 3S. Recently, several turbulence models were studied to characterize the supersonic flow inside the 3S^3,53,54^. The κ-ε turbulence model was not suitable for the description of strong swirl flow^55^. To consider the strong swirl flow inside the 3S, the accuracy of three turbulence models including κ-ε, κ-ω and V2-f were compared, and the most accurate turbulence model was selected for an appropriate description of gas flow through the 3S. The calculated AARD% for the κ-ε, κ-ω, and V2-f turbulence model were about 25.39%, 20.56%, and 8.65%, respectively. In addition, as shown in Fig. 2, the calculation accuracy of the V2-f turbulence model could provide the requirements of engineering applications. Consequently, the V2-f turbulence model was used to describe complex turbulence flow inside the 3S. A new parameter called the elliptic blending function (α) was considered in the V2-f turbulence model. Furthermore, the V2-f turbulence model uses an automatic wall treatment, which switches between wall function formulation and a low-Reynolds-number formulation. This characteristic provides a robust formulation for the V2-f turbulence model.

The V2-f turbulence model employed the Eqs. (20)–(22) to describe the swirl flow through the 3S, as follow:

20 $$\rho \left( {u \cdot \nabla } \right)k = \nabla \cdot \left[ {\left( {\mu + \frac{{\mu_{T} }}{{\sigma_{K} }}} \right)\nabla k} \right] + P_{k} - \rho \varepsilon$$

21 $$\rho \left( {u \cdot \nabla } \right)\varepsilon = \nabla \cdot \left[ {\left( {\mu + \frac{{\mu_{T} }}{{\sigma_{\varepsilon } }}} \right)\nabla \varepsilon } \right] + \frac{1}{\tau }\left( {C^{\prime}_{\varepsilon 1} \left( {\zeta ,\alpha } \right) - C^{\prime}_{\varepsilon 1} \left( {k,\varepsilon ,\alpha } \right)\rho \varepsilon } \right)$$

22 $$\rho \left( {u \cdot \nabla } \right)\zeta = \nabla \cdot \left[ {\left( {\mu + \frac{{\mu_{T} }}{{\sigma_{\zeta } }}} \right)\nabla \zeta } \right] + \frac{2}{k}\left( {\alpha^{3} \mu + \frac{{\mu_{T} }}{{\sigma_{k} }}} \right)\nabla k \cdot \nabla \zeta + \left( {1 - \alpha^{3} } \right)f_{w} + \alpha^{3} f_{h} - \frac{\zeta }{k}p_{k}$$

where *ε, κ* and *ζ* show the turbulence dissipation rate, turbulent kinetic energy, and turbulent relative fluctuations, respectively. The production term (*P*_*k*_) and turbulent viscosity (*µ*_*T*_) in Eqs. (20)–(22) were determined by Eq. (23) and Eq. (24), respectively:

23 $$P_{k} = \mu_{T} \left[ {\nabla u:\left( {\nabla u + \left( {\nabla u} \right)^{T} } \right) - \frac{2}{3}\left( {\nabla \cdot u} \right)^{2} } \right] - \frac{2}{3}\rho k\nabla \cdot u$$

24 $$\mu_{T} = \rho C_{\mu } k\zeta \tau , \, \tau = \max \left[ {\frac{k}{\varepsilon },C_{\tau } \sqrt {\frac{v}{\varepsilon }} } \right]$$

Moreover, the Reciprocal wall distance (*G*) and elliptic blending function (*α*) were calculated using the following equations:

25 $$\alpha - L^{2} \nabla^{2} \alpha = 1, \, L = C_{L} \max \left[ {\frac{{k^{1.5} }}{\varepsilon },C_{\eta } \left( {\frac{{v^{3} }}{\varepsilon }} \right)^{0.25} } \right]$$

26 $$\nabla G \cdot \nabla G + \sigma_{w} G\left( {\nabla \cdot \nabla G} \right) = \left( {1 + 2\sigma_{w} } \right)G^{4} , \quad l_{W} = \frac{1}{G} - \frac{{l_{ref} }}{2}$$

27 $$f_{w} = - \zeta \frac{\varepsilon }{k}, \, f_{h} = - \frac{1}{\tau }\left( {C_{1} - 1 + C_{2} \frac{{p_{k} }}{\rho \varepsilon }} \right)\left( {\zeta - \frac{2}{3}} \right)$$

where *C*_1_, *C*_2_, *C*_μ_, *C*_ε1_, *C*_ε2_, *C*_L_, *C*_τ_, *C*_η_, *σ*_k_, *σ*_ε_, *σ*_ζ_, and *κ*_v_, are the constant in the above equations. The temperature drop through the nozzle was calculated by solving the energy equation and the explained turbulence model simultaneously.

### The line-type of the wall of the 3S

**Figure 3:**
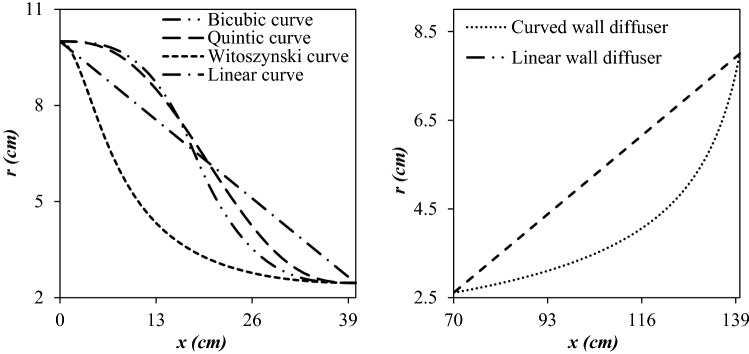
Considered line-type for the convergent section of the Laval nozzle (left) and diffuser (right).

The Laval nozzle is composed of three main sections, including the converging section, the throat, and diverging section. In this equipment, the Laval nozzle plays a crucial role in the liquefaction of heavy hydrocarbons and directly influences the separation efficiency. It is desirable to have higher pressure and lower temperature at the outlet of the Laval nozzle, which causes less pressure loss inside the 3S. The line-type of the convergent section influenced the cooling performance of the 3S^37^. In this study, four different configurations (Linear curve, Witoszynski curve, Bi-cubic curve and Quintic curve) ^57^ were considered for the convergent section (Fig. 3).

The divergent angle (*α*) of the Laval nozzle was determined using the Eq. (28), where subscripts “In”, “Th” and “Out” stand for the inlet, throat and outlet, respectively:

28 $$Tan\left( \alpha \right) = \frac{{r_{Out} - r_{Th} }}{{x_{Out} - x_{Th} }}$$

The *α* = 1° was assumed for all studied structures in this paper. Furthermore, the divergent angle could not be decreased significantly because the length of the 3S becomes very large which is not suitable for practical applications. The role of the diffuser is the transformation of the kinetic energy to pressure energy. If a wrong divergent angle is employed for the diffuser, the boundary layer and shock wave interact with each other, and this can postpone the variation of supersonic flow to subsonic flow. Two different configurations were considered for the diffuser: Liner wall diffuser and curved wall diffuser. The cross-sectional area of the curved wall diffuser can be determined by Eq. (29):

29 $$r = \frac{{r_{1} }}{{\sqrt {1 + \left( {\frac{{r_{1}^{2} }}{{r_{2}^{2} }} - 1} \right)\frac{x}{L}} }}$$

where *r* is the radius at a distance x from the inlet of the diffuser at an arbitrary cross-section, respectively. In addition, *r*_*1*_ and *r*_*2*_ are the radius of the diffuser at the inlet and outlet, respectively. Figure 3 presents the line-type curve of the convergent section and diffuser. It was assumed that the size of the outlet diameter would always be 80% of the inlet diameter.

### Mesh independency

**Figure 4:**
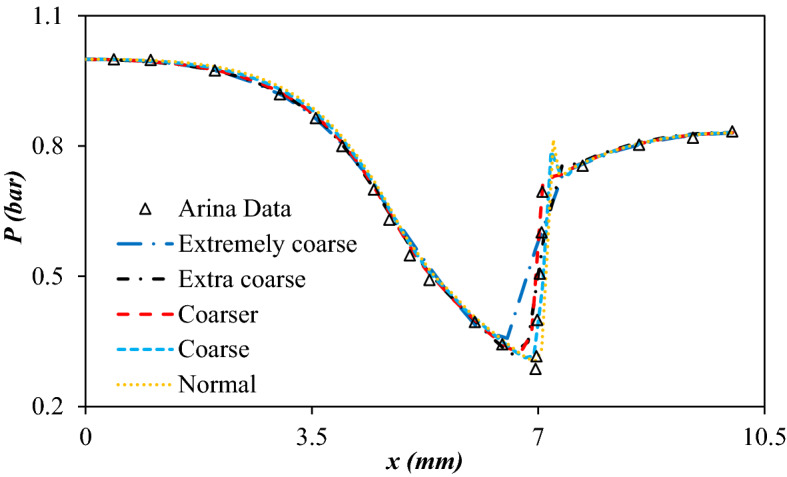
The influence of mesh sizes on the predicted pressure profile.

In this study, the COMSOL Multiphysics^®^ Version 5.4″ software, which solves the developed equations with the finite element method, was used to describe the natural gas flow through the 3S. The computational time was considered equal to 0.06 s for all simulations. In addition, the convergence criterion of 10^–4^ was selected for all developed equations. The Parallel Sparse Direct Linear Solver (PARDISO) solver was employed to solve the time-dependent equations. Moreover, both iterative and direct multi-grid solvers were used in this study. The mesh quality and density have a significant influence on the accuracy of the numerical results. The tetrahedral mesh was used for dividing the studied volume into smaller parts. The pressure profile along the central axis was chosen for the grid independence test. Five types of mesh sizes were considered in this study, including extremely coarse (40,316 cells), extra coarse (71,869 cells), coarser (116,792 cells), coarse (207,927 cells), and normal grids (370,543 cells). Figure 4 shows the influence of grid density on the pressure profile along the central axis of the nozzle. It can be observed that the 207,927 mesh cells and 370,543 mesh cells showed the same result for the pressure profile across the length of the 3S. While the 207,927 mesh cells showed accurate results but the 370,543 mesh cells were selected for exact prediction of shockwave position.

The Grid convergence index (GCI) was also used to further investigate the accuracy of results further and mesh independency. The GCI was calculated by the Eq. (30)^58,59^:

30 $$GCI_{21} = \frac{{F_{s} \left| \varepsilon \right|}}{{r_{21}^{p} - 1}}$$

It can be observed that the GCI of normal mesh size was as follows:

31 $$\frac{{GCI_{32} }}{{r^{p} GCI_{21} }} \approx 1$$

Therefore, it can be concluded that the normal mesh size (370,543 mesh cells) provided the mesh independency.

### Equation of state

To estimate the thermo-physical properties of natural gas in a 3S, an accurate equation of state (EoS) should be considered. The Soave–Redlich–Kwong (SRK), and Peng-Robinson (PR) EoS are generally employed in the oil and gas industry. The prediction of Joule–Thomson inversion curve (JTIC) is a severe test for testing any EoS. Methane is the main component of natural gas. To investigate the capabilities of considered EoS, the calculated JTIC using these EoSs were compared with the experimental data of methane in the previously published works by the authors^4,60,61^. The results demonstrated that the SRK EoS is more accurate than the Van der Waals (VdW) and the PR EoS in predicting the JTIC of pure methane. In addition, in a separate work, the accuracy of several EoS was examined to predict the Joule–Thomson coefficient and the JTIC, and it was found that the SRK EoS is the most accurate EoS in predicting the JTC and JTIC^61^. Consequently, the SRK EoS was selected as the base EoS in this CFD modeling.

### Considered NGL recovery unit

A NGL recovery unit was considered as a research background in this study. The main purpose of an NGL recovery plant is separation of heavy hydrocarbons from the natural gas stream. Industrial data was obtained from a dehydration NGL recovery unit in which works based on the JT processes. This unit is referred to as the three stage system. The JT process efficiency is low due to the low NGL recovery and cooling performance^3^. The 3S process can be employed instead of the JT process due to the higher NGL recovery and cooling performance^3^. This NGL/gas dehydration unit separates the produced fluid from the gas well in a series of separators. The feed gas has a high pressure, thus the first stage separation starts at very high pressure, where the liquid phase is removed from the gas stream. The continuous phase then directed to the medium pressure vessel, where heavier hydrocarbon and remaining water was removed and finally at the third separators more liquid is separated and gas dew point was adjusted. In this paper, the ability of the 3S to recover NGL by using it in the third stage of separation was investigated. The aim of this structure modification is to separate the light hydrocarbon as much as possible at the similar operating condition with the JT process.

## Result and discussion

Proper separation of water vapor and natural gas condensate is crucial from industrial and technical standpoints. The 3S could be employed in the natural gas industry to recover and separate the heavy hydrocarbons and water vapor from the natural gas stream. The principle of the refrigeration process for natural gas dehydration and NGL recovery is that the concentration of condensable components decreases with the decrease of the operational temperature. The condensable component is liquefied in the Laval nozzle during natural gas expansion and then separated at the drainage port due to the strong centrifugal force. The operating condition, separator geometry, and configuration of the swirler characterize the separation performance of each 3S. The developed model will be employed in this section to evaluate the influence of structural and operational parameters on the performance of the 3S.

**Figure 5:**
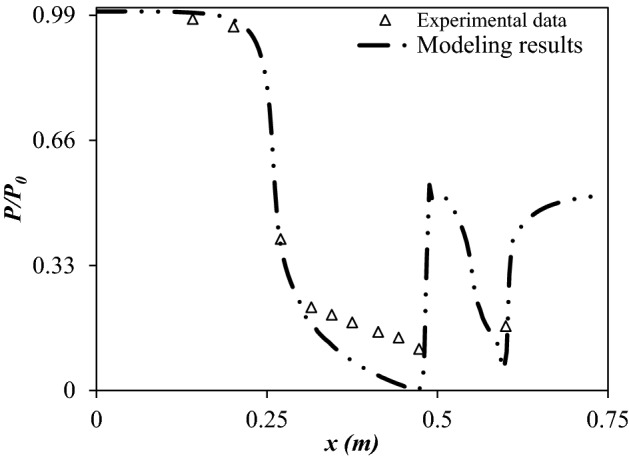
Validation of the developed CFD model by recorded experimental data by Eriqitai et al.^62^.

The model validation has been carried out in this section (Fig. 5). The validity of the developed CFD model was certified by recorded experimental data by Eriqitai et al.^62^. The comparison of simulation results with experimental data of Eriqitai et al., clearly demonstrated that the developed model could be successfully employed for the proper description of separation behavior inside the 3S (Fig. 5).

### The influence of structural parameters

#### Nozzle structure

It is evident that the nozzle structure directly influences the flow behavior and separation efficiency. The purpose of this section is to optimize the nozzle structure using the CFD modeling. To obtain the optimal condition, various structures were considered, and their configuration was optimized based on the average cooling temperature and minimum achievable temperature. The average cooling temperature from the inlet to the outlet of the Laval nozzle was determined by the Eq. (32):

32 $$\delta = \iiint {T_{x} dV}$$

**Figure 6:**
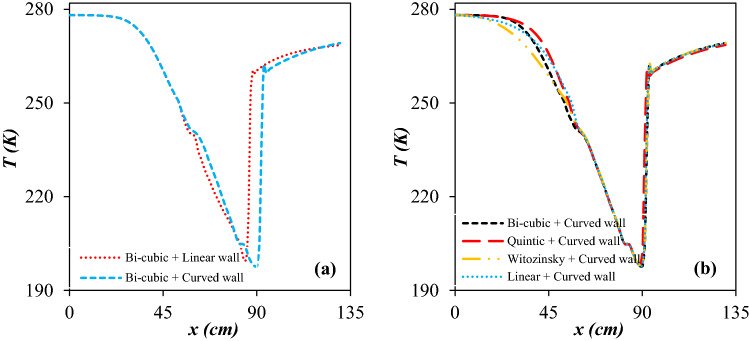
The influence of diffuser line-type (**a**) and convergent section line-type (**b**) on the temperature profile at the center-line of considered 3S.

For all simulations, the boundary conditions of the studied nozzle were fixed. In addition, to optimize the profile curve of the diffuser, the line-type of the convergent section remained constant, and the Bi-cubic analytical formula was employed to design the converging section of the nozzle. The length of the low-temperature region has a significant effect on the size and density of the liquid droplets. Under the condition that geometrical parameter like inlet and outlet diameter is specified (*D*_*In*_ = 11 cm, *D*_*Out*_ = 8.8 cm, *L*_*Laval*_ = 80 cm, and *L*_*Diffuser*_ = 50 cm), the cooling performance is a function of profile curve of the convergent section and diffuser. To investigate the influence of the diffuser profile curve, the temperature distribution along the nozzle axis was presented in Fig. 6a. It can be observed that the diffuser profile curve has a strong effect on the minimum temperature along with the shockwave position for a fixed operational condition. For the curved wall diffuser, the refrigeration zone is longer at the upstream of the shockwave. Consequently, the linear wall diffuser cannot meet the design requirement suitably. Furthermore, for the linear wall diffuser, the velocity profile is not uniform and changes too fast. Therefore, to extend the refrigeration zone and achieve a lower minimum temperature, the curved wall was selected for the diffuser. This decrease in temperature increases the rate of nucleation. In other words, when the natural gas temperature becomes less than the condensation temperature of its constituent, the nucleation will be initiated and then liquid phase will be formed inside the nozzle. In addition, it should be considered, the shockwave position should be located after the drainage point to prevent the re-evaporation of liquid droplets due to the abrupt change in operating temperature. In other words, after crossing the shockwave, the gas temperature increases abruptly which results in re-evaporation of liquid droplets which deteriorates the separator performance.

It is evident that both liquefaction and separation of droplets occur at the Laval nozzle. For this reason, the line-type of the convergent section of the Laval nozzle should be optimized. Then, to optimize the line-type of the convergent section, the profile curve of the diffuser was considered fixed and included with the mentioned curved wall formula (Fig. 6b). The convergent section line-type can be designed using different methods. Figure 6b shows the temperature distribution inside the nozzle when the convergent section was designed by Linear, Bi-cubic, Witoszynski and Quintic curve formula, respectively. Simulation result illustrated that the shockwave position for different line-type of the convergent section were identical. Therefore, the value of minimum temperature by the Witoszynski curve was almost the same as the Linear, Bi-cubic and Quintic curve. Contrary to this, the Witozinsky curve generated a longer low temperature domain inside the 3S. Consequently, to extend the refrigeration zone, the Witoszynski line-type was selected for the convergent section of 3S. Furthermore, simulation results demonstrated that the temperature and pressure variation in the convergent section designed by the Witoszynski curve is more uniform and changed smoothly compared to other considered line-types. As stated in the previous paragraph, this decrease in temperature and increase in refrigeration zone enhances the rate of nucleation and thus increases the size of the liquid droplets formed. Therefore, the increase of liquid droplet size leads to the improvement of separation performance^4,7^. This conclusion is in agreement with previously published work^37^.

**Figure 7:**
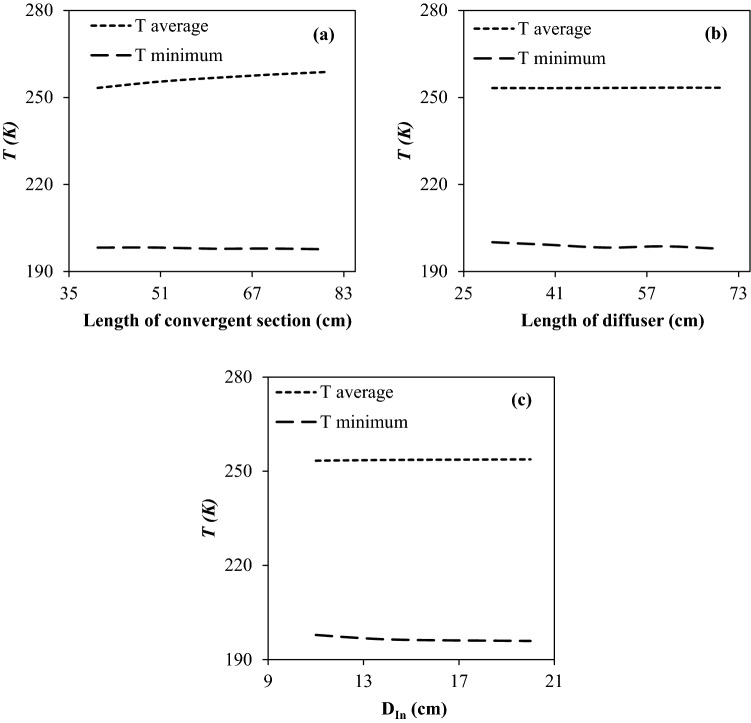
The influence of (**a**) length of convergent section, (**b**) length of diffuser and (**c**) inlet diameter of nozzle on the minimum temperature and average cooling temperature.

The average cooling temperature and minimum temperature play a crucial role in the NGL recovery rate at various operational conditions. The influence of the length of convergent section and diffuser on the average cooling temperature and minimum temperature was investigated while other geometrical parameters remain fixed. It can be observed that the lowest average cooling temperature was obtained for the shortest convergent length. Furthermore, the simulation results (Fig. 7a) illustrated that the minimum temperature for various convergent lengths are similar. Therefore, the optimal value of convergence length was about 40 cm.

Then, the influence of diffuser length and nozzle inlet diameter on the minimum temperature and average cooling temperature were investigated. The simulation results showed that the diffuser length and inlet diameter of the nozzle did not have a significant effect on the average cooling temperature (Fig. 7b,c). Contrary to this, as the diffuser length and inlet diameter of the nozzle increase, the minimum temperature decreases further. Consequently, the optimal dimensions (L_Conv_ = 40 cm, L_Laval_ = 60 cm, D_In_ = 20 cm, D_Out_ = 16 cm, L_Diffuser_ = 70 cm) were obtained based on the minimum temperature and average cooling temperature. The minimum temperature and maximum velocity inside the optimal nozzle are about 195.9 K and 508.5 m/s, which is suitable for liquefaction of condensable components in the natural gas like water vapor and heavy hydrocarbons.

### Passive method of generation of swirl flow

The swirler is one of the main components of the 3S. In this section, the influence of the structure of the swirler on the flow characteristic and separation efficiency was investigated. Generally, the swirler is divided into active and passive swirler. In the conventional 3S (3S equipped with the active swirler), the swirler is located at the convergent section of the nozzle. For the active method, the cross-sectional area of natural gas is decreased due to the existence of the swirler. These swirler lead to the disturbance of the flow field and degradation of cooling performance. In order to overcome these deficiencies, three new structures based on the passive method were proposed for the generation of swirl motion. It was expected that this method decreased the energy loss and improved the cooling performance of the 3S.

#### 3S equipped with side injected swirler

**Figure 8:**
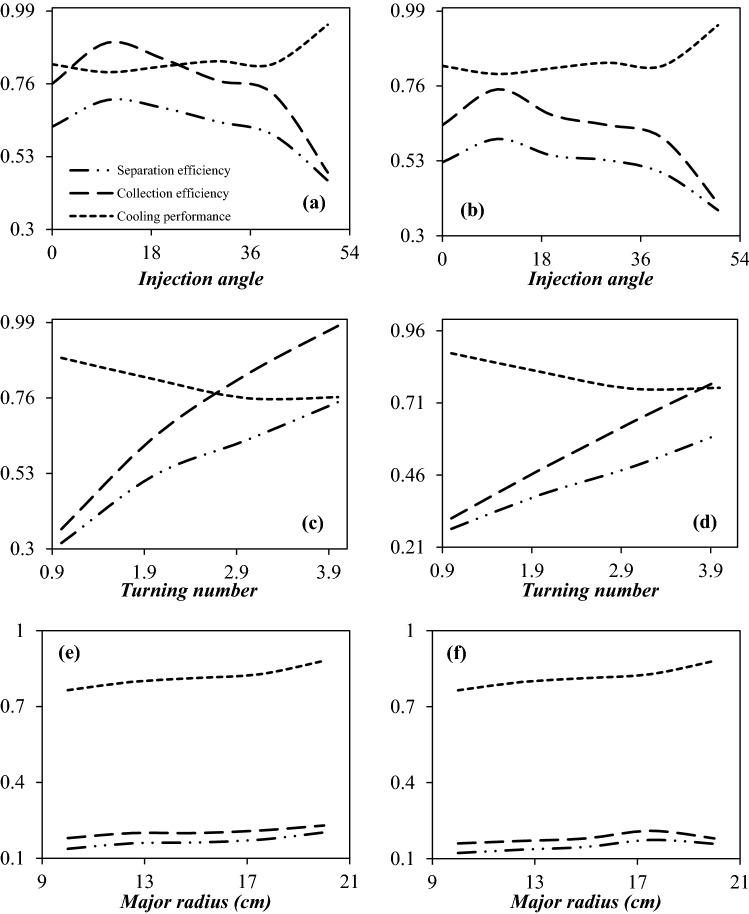
The influence of structural parameters on the collection efficiency, cooling performance and separation efficiency of 3S quipped with side injected swirler (**a**,**b**), serpentine swirler (**c**,**d**) and U-turn swirler (**e**,**f**) for water (**a**,**c**,**e**) and hydrocarbon droplets (**b**,**d**,**f**).

Figure 8a,b shows the cooling performance, collection efficiency, and separation efficiency of the 3S equipped with side injected swirler under the condition that the injection angle (tangential inlet to a cross section) is 0°, 10°, 20°, 30°, 40°, and 50° respectively. It can be observed that for a fixed operational condition when the injection angle increased from 0° to 50°, the location of the shockwave shifted from x = 54.4 cm to x = 60 cm. The reason for this is that by changing the injection angle from 0° to 50°, the loss of pressure energy is reduced. It is clear that the higher the pressure energy of the passing fluid, the more the shockwave position moves towards the output. This change in the position of the shockwave also leads to lower temperatures and an improvement in the cooling performance. In addition, it can be observed that increasing the injection angle from 0° to 10° increased the minimum temperature inside the nozzle and deteriorated the cooling performance; while this modification improved the collection efficiency. For the injection angle of 10° and 50°, the minimum temperatures were about 215.78 K and 204.02 K, respectively. It means that the cooling performance is improved about 18.8% for an injection angle of 50° compared to an injection angle of 10°. Contrary to this, for the injection angle of 10° and 50°, the collection efficiency of water droplets were about 89% and 48%, respectively. In conclusion, the trade-off between the cooling performance and collection efficiency should be found. Figure 8a,b show that the separation efficiency of the nozzle firstly improved and then deteriorated with the increase of the injection angle. When the injection angle increases from 10° to 50°, the separation efficiency of water droplets decreases from 70.8 to 45.5%, indicating that after a certain degree, the separation efficiency is no longer increased with increasing the injection angle. It can be found that at the swirl angle of 10°, the highest separation efficiency was obtained. Consequently, the injection angle of 10° was proposed for the 3S, based on the complete consideration of cooling performance and collection efficiency. For all injection angles from 0° to 50°, a certain amount of pressure drop was considered (certain pressure energy) for NGL recovery. It is clear that the more pressure energy is expended in generation of swirl motion (and thus creating a centrifugal force), the less energy can be expended in cooling. On the other hand, the higher the swirl flow and the centrifugal forces, the higher the collection efficiency. As shown in Fig. 10, the injection angle of 10° provides more swirl motion, and based on the results, the highest collection efficiency occurs at this angle. Therefore, the lowest cooling efficiency at the injection angle of 10° compared to other injection angles is due to the fact that the energy available to reduce the temperature is less. But in the injection angle of 10°, the effect of collection efficiency is more than cooling performance and as a result, the highest separation efficiency is obtained in this angle.

#### 3S equipped with serpentine swirler

The influence of various structural parameters of the 3S equipped with serpentine swirler such as serpentine radius, axial pitch, and the number of turns (turning number) on the separation efficiency were investigated. Initially, a sensitivity analysis was conducted on the influence of the serpentine radius and axial pitch on the cooling performance, collection efficiency, and separation efficiency. The studied ranges of serpentine radius and axial pitch were 10–20 cm (R_big_ = 10, 12.5, 15, 17.5, and 20 cm) and 12–18 cm (12, 14, 16, and 18 cm), respectively. Based on the simulation results, in the serpentine radius of 20 cm and the axial pitch of 16 cm, the highest separation efficiency was obtained. Therefore, the axial pitch of the 3S equipped with a serpentine swirler has to be moderate for higher separation efficiency. For smaller serpentine radius, a large curvature is observed in the swirler, while for larger serpentine radius, a slight curvature is observed. This behavior means that for small swirler, the role of drag force is more significant than the centrifugal force, while for large swirler, the role of centrifugal force is more significant than the drag force.

Figure 8c,d show the cooling performance, collection efficiency, and separation efficiency of 3S under the condition that the number of turns were 1, 2, 3, and 4, respectively. It can be observed that while an enhancement of the number of turns improved the collection efficiency but, this variation had an adverse influence on the cooling performance of the nozzle. In conclusion, increasing the number of turns had both positive and negative influences on the separation efficiency of the 3S. However, the effect of collection efficiency on the separation efficiency for this case was more significant than cooling performance. Consequently, it was proposed that the number of turns to be 4 to achieve suitable separation efficiency.

#### 3S equipped with U-turn swirler

In this section, the flow characteristics and separation performance of the 3S equipped with a U-turn swirler were studied. In this structure, the required centrifugal force was generated by a U-turn tube with a constant cross-sectional area. The influence of U-turn radius on the expansion characteristic (cooling performance) and collection efficiency was plotted in Fig. 8e,f. It can be observed that, when the U-turn radius increased from 10 to 20 cm, the separation efficiency of water and condensate droplets increased from 13.7 to 20.2% and 12.2 to 15.8%, respectively. This conclusion indicated that the U-turn radius had little influence on the separation efficiency of 3S equipped with a U-turn swirler. This U-turn swirler showed suitable cooling performance, while the collection efficiency of this configuration was poor.

#### The influence of swirler type on the swirl velocity

**Figure 9:**
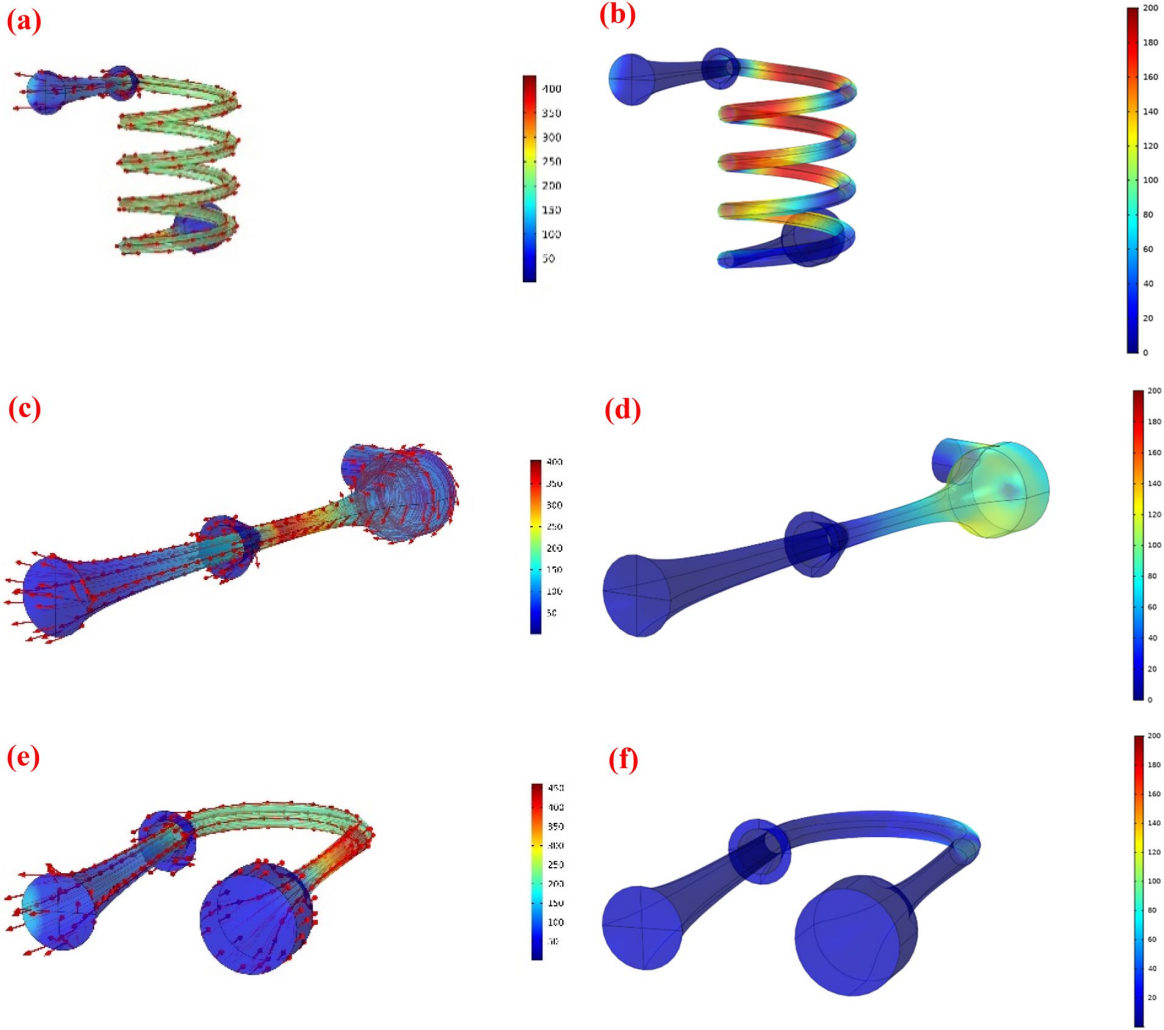
Velocity profile (m/s) (**a**,**c**,**e**) and swirl velocity distribution (m/s) (**b**,**d**,**f**) for various swirler structures.

The simulation results presented in the previous section demonstrated that the structure of the swirler significantly affected the separation efficiency of the nozzle. In addition, by comparing Figs. 8 and 9, it can be concluded that at the higher swirl strength, the collection efficiency was significantly improved. Contrary to this, with the increase of swirl intensity, the minimum temperature was raised, causing a lower rate of liquid droplet formation and separation efficiency. On the one hand, a significant centrifugal force is required, on the other hand, this swirl flow destroys the cooling performance of the Laval nozzle. Therefore, there is an inverse relationship between swirl intensity and cooling performance. Based on this, a suitable point should be found where both the collection efficiency and the cooling performance are in their optimal conditions. Furthermore, it is evident that as the maximum swirl velocity increases, the centrifugal force increases. Consequently, the serpentine swirler provides a stronger centrifugal force than others. Furthermore, with the increase of swirl intensity, the non-uniformity of velocity profile along radial direction becomes more obvious and the velocity gradient raised close to the separator wall. As shown in Fig. 8, the 3S equipped with serpentine and U-turn swirler provided non-uniform velocity profiles compared to the 3S equipped with the side injected swirler.

#### Comparison between three different swirler

Based on the obtained results (Fig. 8), it can be concluded that the U-turn swirler provides lower separation efficiency compared to the serpentine and side injected swirler under the same operating condition. In conclusion, the side injected and serpentine swirler induced a stronger centrifugal force to separate the liquid droplets from the gas phase. The side inject and serpentine swirler at the optimal point had similar separation efficiencies (about 70%). The serpentine swirler generated a centrifugal force inside the 3S stronger than the side injected swirler. While a great swirl flow was generated by a serpentine swirler, but this structure deteriorated the expansion characteristic and the cooling performance of the 3S separator. Furthermore, it can be seen that the velocity profile was not uniformly distributed for the serpentine swirler. The main disadvantage of a serpentine swirl generator is its low stability due to the generation of high turbulency. Consequently, for the optimal structure, the swirling generator is mounted in the nozzle entrance. Therefore, based on the cooling performance and flow stability, the side injected swirler was selected as the most optimal case for further studies. On the other hand, installing a side injected swirler for industrial applications is easier and more economical than the installation of the serpentine swirler.

#### Side injected swirler

Only a few studies consider the influence of swirler structure on the flow characteristic. Furthermore, almost all of these studies are focused on the active swirler. For example, Yang et al.^47^ used the CFD modeling to optimize the vane structures in the 3S for gas purification application. They observed that strengthening the swirl flow weakens the expansion characteristic. Furthermore, they optimized the swirl angle, height and number of the static vane by numerical analysis. Wen et al.^21^ employed the DPM to evaluate the influence of delta wing on the strong swirl flow. They observed that the large delta wings generate a strong swirl flow, while the small delta wings have minor effects on the flow above it. For the active method, the presence of swirler in the middle of gas flow may deteriorate the expansion phenomenon and produce some residence for the flow of natural gas inside the nozzle. Furthermore, the active swirler not only causes the energy loss but also makes the natural gas flow more turbulent. For example, Wen et al.^63^ numerically studied the influences of swirls on the natural gas velocity, mass flow rates and temperature in the 3S. They reported that increase in swirl strength caused non-uniform radial distribution of the gas Mach number at the outlet. Therefore, to overcome these deficiency, a new method of generation of swirl flow named as passive method was considered in this study. In this study, several alternative structures for swirler were proposed and their influence on the flow behavior was evaluated.

The configuration of the swirler plays a crucial role in the separation performance of the 3S. In this paper, new structures for swirler were proposed and then examined the influence of them on the separation performance of the 3S. In view of improving the energy loss, three passive methods for generation of swirl flow were considered to overcome the problem of excessive resistance. In these swirler, the swirl motion was generated by passive methods such as side injected swirler, U-turn swirler and serpentine swirler. To improve the nozzle separation performance, the swirl flow behavior inside the nozzle was optimized. As seen in the previous sections, the side injection pipe generates a strong centrifugal force to separate the liquid droplets from the gas stream. Therefore, in this paper, instead of using an active swirler, a new method called side injected swirler was used for generation of swirl flow.

#### Swirl velocity and dimensionless mass flow rate in 3S equipped with side injected swirler

**Figure 10:**
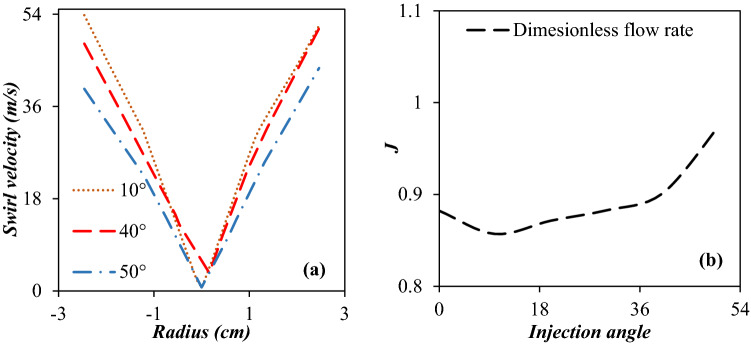
Swirl velocity (at y (length) = 0.4 m) (**a**) and mass flow ratio (**b**) rate for various injection angles.

Figure 10a presents the profiles of the swirl velocity at y = 0.4 m for six different considered injection angles, including 0°, 10°, 20°, 30°, 40° and 50°, respectively. As illustrated in Fig. 10a, the swirl velocity increased and reached its maximum values at the injection angle of 10°, and then decreased. The maximum swirl velocities for injection angle of 10° and 50° were about 53.8 m/s and 43.5 m/s, respectively. Therefore the swirl angle of 10° generated a higher centrifugal force for the separation of liquid droplets from the natural gas stream. Consequently, the designed 3S (3S equipped with side injected swirler) provided suitable swirl velocity and separation efficiency without employing any external equipment.

Figure 10b depicts the ratio of the total mass flow rate of the 3S equipped with the swirler to the mass flow rate of the simple structure (J = m_Swirl_/m_Simple_), versus the injection angle. As can be seen, the minimum mass flow rate was obtained for an injection angle of 10°. This can be justified based on swirl strength. It can be observed that as the swirling strength enhances, the total mass flow rate at the throat decreases. Because at this angle more energy is spent on creating swirl motion and less energy is spent on moving forward. Therefore, the mass flow rate decreased.

### The structure of drainage port

**Figure 11:**
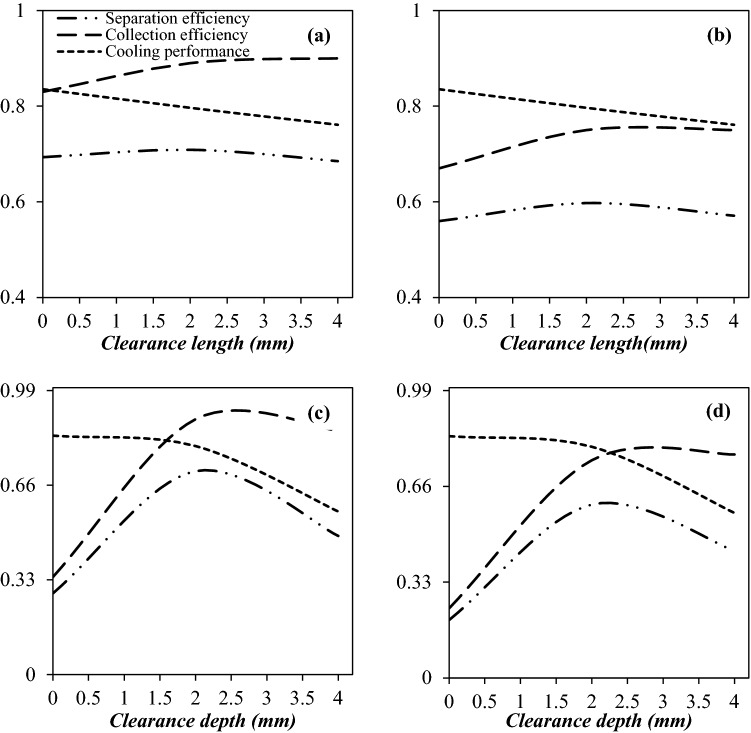
The influence of clearance length (**a**,**b**) and clearance depth (**c**,**d**) on the separation efficiency of water droplet (**a**,**c**) and condensate droplet (**b**,**d**).

In this section, our purpose is to optimize the structure of the drainage port. For all cases, the slip gas flow rate is about 10% of the total flow rate through the nozzle. The effect of clearance length on the separation efficiency of water droplets and condensate droplets are presented in Fig. 11a,b, respectively. Three different clearance lengths were considered for the drainage system. The investigated clearance lengths were 0 mm, 2 mm, and 4 mm (at constant clearance depth of 2 mm), respectively. It can be observed that the separation performance was influenced by the clearance length of the drainage point. As shown in Fig. 11a,b, when the clearance length increased, the collection efficiency also increased, and the cooling efficiency decreased. Therefore, the clearance length should be optimized to achieve a trade-off between the collection efficiency and cooling performance. Simulation results demonstrated that when the gap size increased from zero to 4 mm, the collection efficiency of the water droplets and condensate droplets improved from 83 to 90% and 67 to 75%, respectively. The cause of this improvement in collection efficiency is that more liquid droplets can directly enter into the drainage port and then be removed from the gas phase with the larger gap size. Conversely, the longer the clearance length, the greater the pressure energy loss and consequently the lower the cooling performance. In addition, it can be observed that when the clearance length increased from zero to 4 mm, the separation efficiency of the nozzle firstly improved and then decreased. Therefore, the highest separation efficiency was obtained at the clearance length of 2 mm. Consequently, based on the obtained results, the final clearance length was selected as 2 mm.

Figure 11c,d present the effect of clearance depth of the drainage port on the separation efficiency. The investigated clearance depths were zero (flush type), 2 and 4 mm (at constant clearance length of 2 mm), respectively. It can be observed that increasing the size of clearance depth has both positive and negative effects on the nozzle performance. Simulation results demonstrated that as the size of clearance depth increased from 0 to 4 mm, the cooling performance of the separator decreased from 83.2 to 56.8%, which means that the nozzle equipped with flush type drainage port was more suitable for nucleation of condensable components. This issue was due to the decrease in cross-sectional area and disturbance in the flow field. Therefore, for a nozzle equipped with flush type drainage structure (L_depth_ = 0 mm), the minimum temperature is lower than a nozzle equipped with an internal extension structure (L_depth_ > 0 mm). The main disadvantage of a nozzle equipped with a flush-type drainage structure was the low collection efficiency. In order to overcome this deficiency of flush type drainage structure, the internal extension structure was installed, and its influence on collection efficiency was investigated. For the internal extension structure, due to the colliding droplet with the inserted wall of the drainage port, the number of separated droplets boosted, which resulted in the improvement of collection efficiency. For instance, under the PLR of 0.2, the collection efficiency of internal extension structure with a clearance depth of 2 mm for condensate droplets is 2.2 times higher than flush type structure. Therefore, the internal extension structure minimized the number of escaped droplets from the separator. The reason for this is that the height of internal extension structure is higher than flush type structure, and as a result, the collection efficiency is improved due to the droplets hitting the wall of the drainage port. Contrary to this, as mentioned previously, for this structure, the cooling performance deteriorated compared to the 3S equipped with flush type drainage port. Therefore, it is necessary to find the optimal point where the highest separation efficiency is obtained. Figure 11c,d show that the highest separation efficiency was obtained for nozzle equipped with an internal extension structure with a clearance depth of 2 mm. In conclusion, based on these criteria, both the clearance length and depth of 2 mm were selected.

### The influence of operating parameters

It is crucial to understand how natural gas will behave under a wide range of operational conditions. In this section, for a nozzle whose structure was optimized in previous sections, the effect of various operational parameters, such as droplet diameter, droplet density, and PLR, on the separation efficiency were examined.

#### The influence of liquid droplet size and density

**Figure 12:**
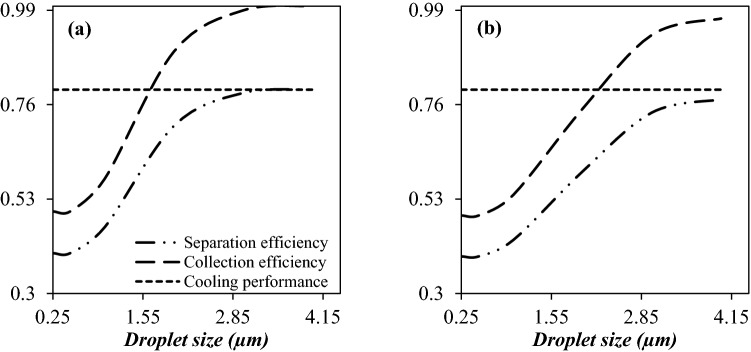
The influence of water (**a**) and condensate (**b**) droplet size on the separation efficiency.

Six different sizes, including 0.25, 0.5, 1, 2, 3, and 4 µm, were defined for the investigated liquid droplets, and the effect of increasing the size and density of the water and hydrocarbon droplets were investigated (Fig. 12). Simulation results showed that the liquid droplets with smaller size and density carried over by the gas phase. It can be observed that the collection efficiency improved with the increase of the liquid droplet size. According to Newton's law of mechanics, the force exerted on larger liquid droplets is greater than on smaller liquid droplets^7^. For instance, for the condensate droplet with a diameter of 0.5 µm, the collection efficiency was about 49%, which means that small liquid droplets escaped easily with the gas phase. Contrary to this, the collection efficiency reached 97% for the same droplets with a diameter of 4 µm. Therefore, the liquid droplet diameter has to be increased for suitable separation performance. To increase the rate of nucleation at the Laval nozzle, the super-saturation degree in the nozzle should be enhanced. After that, the tiny droplets coalesced into each other to form larger droplets that were thrown to the separator wall due to the centrifugal force.

The separation efficiency was also improved as the density of liquid droplets increased. The cause of this improvement is that the liquid droplets are separated from the gas phase due to the gas–liquid density difference. Enhancing this difference improved the separation efficiency. Accordingly, as shown in Fig. 12, all sizes of water droplets have higher separation efficiencies than condensate ones. This can be attributed to a greater centrifugal force for water droplets.

#### The influence of PLR on the separation efficiency

**Figure 13:**
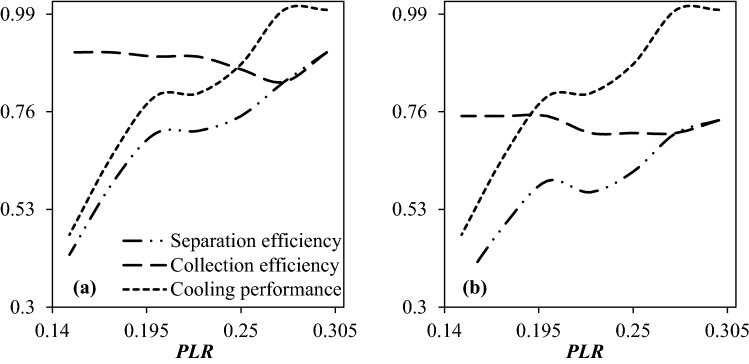
The influence of PLR on the separation efficiency of water (**a**) and condensate (**b**) droplet.

The PLR is one of the important operational parameters which influences the shockwave position, separation efficiency, mass flow rate, and minimum temperature inside the nozzle. Different values of PLR, such as 0.15, 0.175, 0.2, 0.225, 0.25, 0.275, and 0.3, were considered in the optimization scheme to obtain the optimum cooling performance, collection efficiency, and separation efficiency. Figure 13 provides the influence of PLR on the cooling performance, collection efficiency, and separation efficiency of the considered nozzle. The cooling performance of the 3S improved significantly by increasing the PLR. The reason for this is that as the pressure gradient between the inlet and outlet increases, more pressure energy is available to reduce the temperature of the working fluid. Contrary to this, the collection efficiency decreased by increasing the PLR. This decrease in collection efficiency is due to the fact that with increasing the pressure gradient, the velocity of the working fluid increases and as a result, the escape of liquid droplets enhances. But between these two parameters, cooling performance has a more significant effect than the collection efficiency and as a result, the separation efficiency improved with increasing the PLR. CFD modeling demonstrated that the optimal separation performance was obtained at the PLR of 0.3. On the other hand, it should be noted that although increasing the PLR improved the cooling efficiency and separation efficiency, but the amount of energy loss increased significantly.

**Figure 14:**
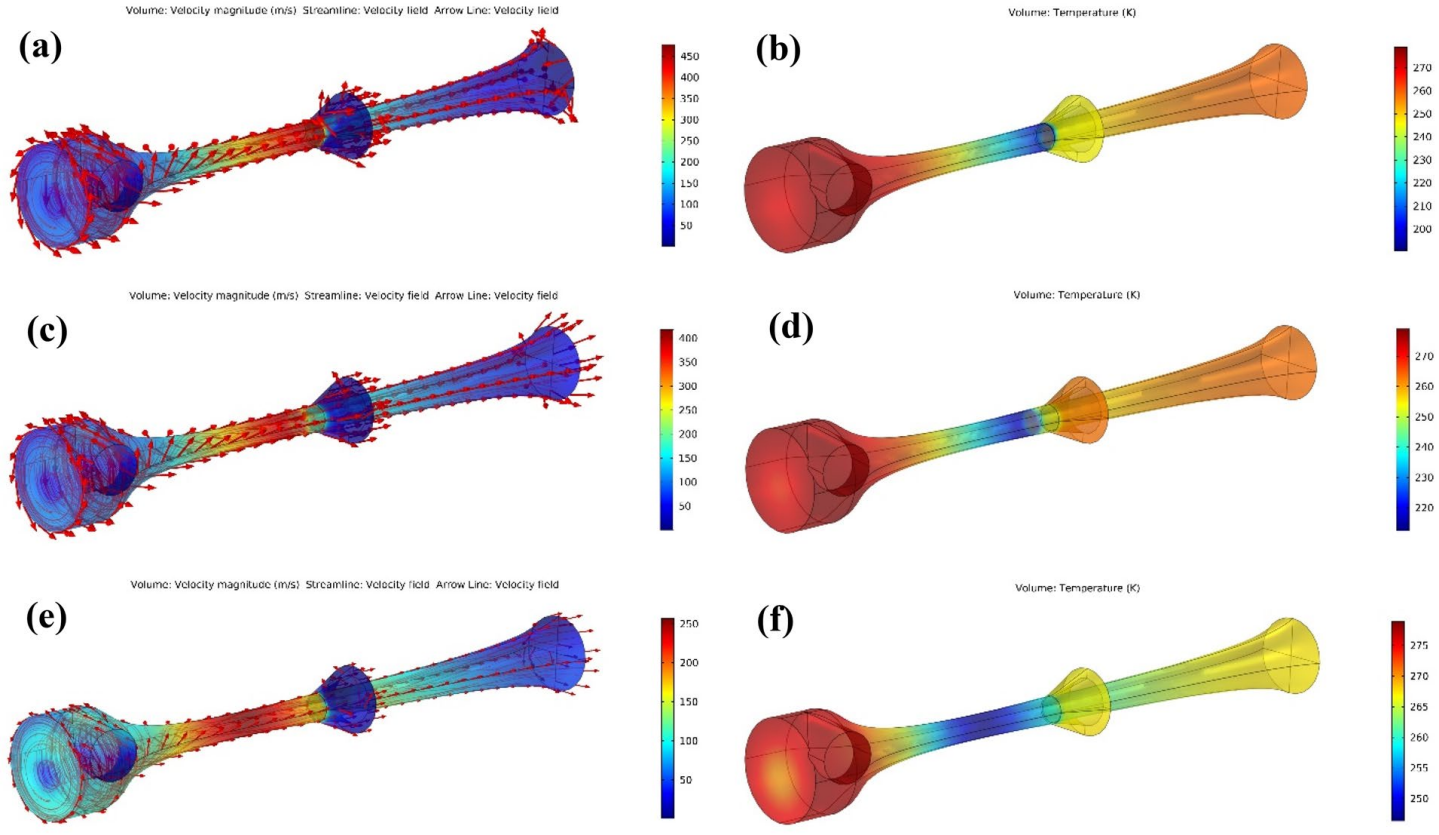
The influence of PLR on the velocity (**a**,**c**,**e**) and temperature profile (**b**,**d**,**f**) for (**a**,**b**) PLR = 0.3, (**c**,**d**) PLR = 0.225 and (**e**,**f**) PLR = 0.15.

The influence of various PLR on the velocity and temperature profile were also studied. As shown in Fig. 14 the supersonic region in the convergent-divergent nozzle enlarged as the PLR increased. Furthermore, the maximum velocity also increased with the increase of the PLR. In contrast, the minimum temperature and the outlet temperature decreased with increasing the PLR, which illustrated that the cooling depth and liquefaction ability improved as the PLR increased.

As shown in Fig. 14, the temperature decreased, and velocity increased abruptly after shock wave occurrence. By the increase in the PLR, the shockwave shifted toward the separator outlet where it surpasses the collection point, whereas decreasing the PLR causes shifting the shockwave toward the nozzle inlet. In case of decreasing the PLR, a portion of the liquid phase is re-evaporated before separation from the gas phase. Therefore, the shockwave position considerably influences the separator performance^64^. For aligning the shockwave position with the collection point, the PLR (or outlet pressure at constant feed pressure) should be varied. For example, when the PLR was decreased to 0.15, the shockwave position moved toward the upstream with the minimum temperature of 246.3 K. In this condition, the Mach number at the throat became less than unity, and the flow condition will not be supersonic.

### Thermodynamic analysis

**Figure 15:**
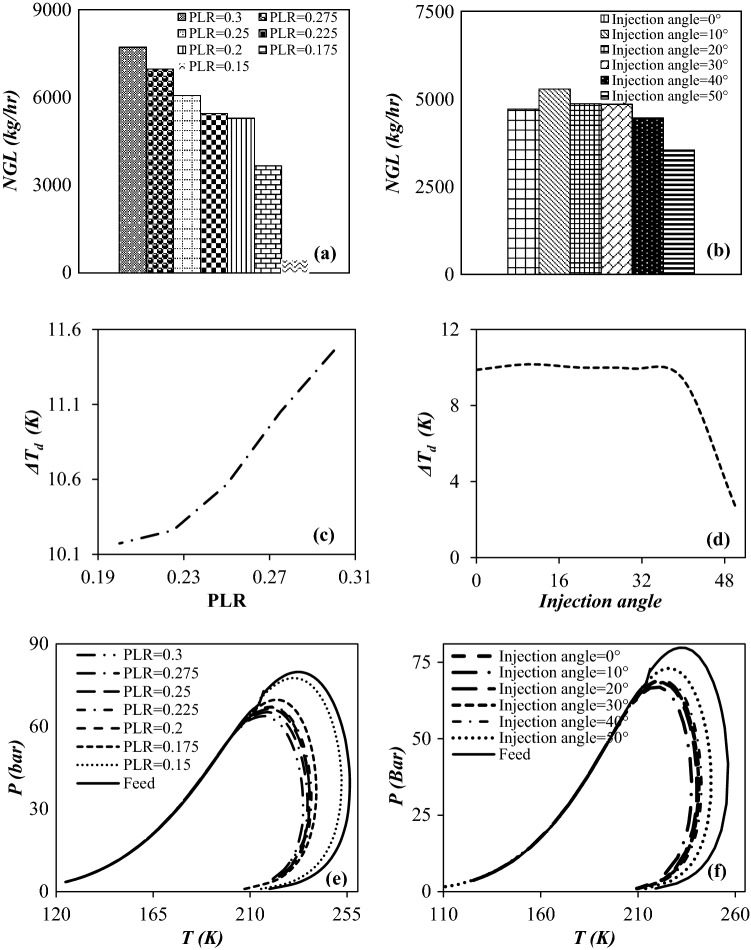
The influence of PLR (**a**,**c**,**e**) (at injection angle of 10°) and injection angle (**b**,**d**,**f**) (at PLR of 0.2), on the (**a**,**b**) rate of NGL recovery, (**c**,**d**) dew point depression and (**e**,**f**) phase envelop diagram of dry gas outlet (entrainment effect was considered).

In Fig. 15, the variation of the phase envelope diagram, rate of NGL recovery, along with the dew point depression were presented as function of PLR and values of injection angles. The HC dew point is defined as the temperature at which the liquid phase will be formed during the cooling process. Figure 15 illustrates that lowering the outlet pressure caused the dew point depression and increase of NGL recovery. For example, for PLR of 0.2 and 0.3, the HC dew point depression was about 10.2 °C and 11.4 °C, respectively. Consequently, the PLR should be adjusted to achieve the desired dew point at the outlet. In addition, it can be observed that for a specified PLR (PLR = 0.2), the maximum dew point depression occurred at the injection angle of 10°. The reason for this issue is that in this injection angle, as shown in “Passive method of generation of swirl flow” section, lower entrainment was observed than other investigated injection angles. Another critical parameter that should be characterized is the phase envelope diagram. The phase envelope diagram at different operating conditions was depicted for the feed gas and dry gas outlet in Fig. 15e,f. The phase behavior of natural gas is a strong function of its components^3^. This diagram for the present gas mixture was plotted based on the SRK EOS. It is clear that the dew point curve is moved to a lower temperature for the dry gas outlet compared to the feed gas. The cause of this phenomenon is that the domain of the phase envelope diagram is a strong function of the heavy fraction. It should be noted that as the performance of the 3S in the separation of heavy hydrocarbons becomes higher, the domain of the phase envelope diagram of the dry gas out becomes smaller^3^. Based on this, the 3S with an injection angle of 10° has the highest separation efficiency.

It can be observed that the presence of heavy hydrocarbons significantly influenced the HC dew point and phase envelope diagram of natural gas. It is evident that as the HC dew point decreases, the gas condensate production rate increases. Figure 15a illustrates that for a specified injection angle (10° for this case), more liquid phase was separated at higher PLR, which means that the condensation efficiency was improved by decreasing the outlet pressure at a specified inlet pressure. In addition, Fig. 15b shows that for a given PLR, the maximum condensate recovery was obtained at an injection angle of 10°. Therefore, based on the analysis of three different parameters, including phase envelope diagram, dew point depression, and rate of NGL recovery, the optimal point for separating gas condensate is at an injection angle of 10° and a PLR of 0.3.

**Figure 16:**
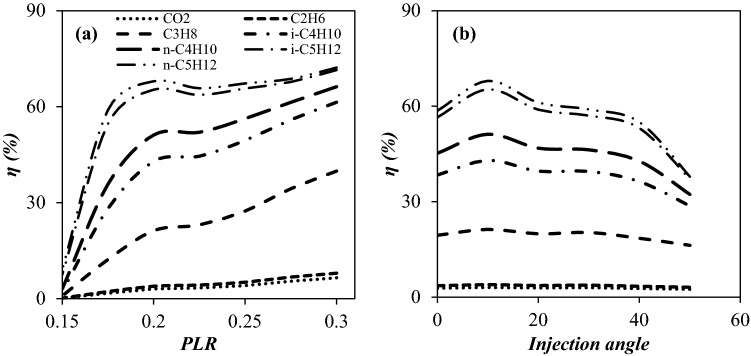
The influence of PLR at injection angle of 10° (**a**) and injection angle at PLR = 0.2 (**b**) on the component collection efficiency.

Another important parameter that can be used to characterize the performance of a 3S is component collection efficiency. Therefore, considering the entrainment effect, the collection efficiency of various components against the PLR and injection angle were depicted in Fig. 16. As can be seen for the injection angle of 10°, with increasing PLR, the collection efficiency of all components, especially heavy hydrocarbons, increased dramatically. In addition, for a given PLR, the maximum component collection efficiency (for all considered components) was obtained at an injection angle of 10°. It is evident that when the heavy hydrocarbon reached the dew point line (liquefaction temperature), they condensed in the 3S. As can be seen, for a specified PLR, the heavier hydrocarbons showed higher component collection efficiencies. This is because these heavier hydrocarbons liquefied at higher temperatures. In addition, for a given hydrocarbon, higher component collection efficiencies at higher PLR was obtained due to the achievement of lower minimum temperatures inside the 3S in this condition.

## Conclusion

In this work, COMSOL Multiphysics software was used to solve the developed equation using the finite element method. The simulation results agree well with the experimental data from literature which demonstrates that the developed model can predict the supersonic separation behavior accurately. Based on the obtained results the line-type of convergent section significantly influences the cooling performance of the 3S. The Laval nozzle designed by Witozinsky curve shows the best cooling performance compared to others. Passive methods for generating swirl flow in a specified 3S was investigated by the CFD modeling. For this purpose, three different structures, including side injected, serpentine, and U-turn, were considered for the swirler. In order to exactly analyze the separation efficiency of the 3S, the collection efficiency and cooling performance was determined for each structure. Based on the performed analysis, 3S equipped with serpentine and side injected structures have similar separation efficiencies. However, the side injected structure with an injection angle of 10° has a higher flow stability and is easier and more economical to build this structure from an economic point of view. Therefore, the 3S equipped with a side injected swirler was selected as the most optimal case.

For the optimized nozzle, the effect of operational parameters such as droplet size, density and the PLR value on the separation efficiency were investigated. It was observed that the larger the size and heavier the density of a droplet, the better the separation efficiency. On the other hand, it was observed that at higher PLR, higher separation efficiency was obtained. The effect of these operational parameters on the rate of NGL recovery, dew point depression, and phase envelope diagram was also investigated for the 3S equipped with a side injected swirler. The simulation results showed that for this case, at higher PLR and at the injection angle of 10°, the rate of NGL recovery and dew point depression were increased.

## Data Availability

The datasets used and/or analysed during the current study available from the corresponding author on reasonable request.
